# Real-life experiments in supermarkets to encourage healthy dietary-related behaviours: opportunities, challenges and lessons learned

**DOI:** 10.1186/s12966-023-01448-8

**Published:** 2023-06-20

**Authors:** Christina Vogel, Coosje Dijkstra, Marlijn Huitink, Preeti Dhuria, Maartje P Poelman, Joreintje D Mackenbach, Sarah Crozier, Jacob Seidell, Janis Baird, Kylie Ball

**Affiliations:** 1 Centre for Food Policy, City, University of London, Northampton Square, London, EC1V 0HB UK; 2 Medical Research Council Lifecourse Epidemiology Centre, University of Southampton, Southampton General Hospital Tremona Road, Southampton, SO16 6YD UK; 3 National Institute for Health Research Southampton Biomedical Research Centre, University of Southampton and University Hospital Southampton NHS Foundation Trust, Southampton, SO16 6YD UK; 4 Department of Health Sciences, Faculty of Science, Vrije Universiteit Amsterdam, Amsterdam Public Health research institute, De Boelelaan 1085, Amsterdam, 1081 HV the Netherlands; 5 Chair group Consumption and Healthy Lifestyles, Wageningen University & Research, P.O. Box 8130, Wageningen, 6700 EW The Netherlands; 6 Department of Epidemiology and Data Science, Amsterdam UMC, location VUmc, Amsterdam Public Health research institute, De Boelelaan 1089a, 1081HV, Amsterdam, the Netherlands; 7 Institute for Physical Activity and Nutrition Research, School of Exercise and Nutrition Sciences, Deakin University, 1 Gheringhap Street, Geelong, VIC 3220 Australia

**Keywords:** Supermarkets, Public-private partnerships, Diet, Food environments

## Abstract

**Background:**

Supermarkets are the primary source of food for many people yet their full potential as a setting to encourage healthy dietary-related behaviours remains underutilised. Sharing the experiences from research groups who have worked with supermarket chains to evaluate strategies that promote healthy eating could improve the efficiency of building such relationships and enhance the design quality of future research studies.

**Methods:**

A collective case study approach was used to synthesise experiences of engaging and sustaining research collaborations with national supermarket chains to test the effectiveness of health-focused in-store interventions. The collective narrative covers studies conducted in three high-income countries: Australia, the Netherlands and the United Kingdom.

**Results:**

We have distilled our experiences and lessons learned into six recommendations for conducting high quality public health research with commercial supermarket chains. These include: (i) ﻿using personal contacts, knowledge of supermarket activities and engaging executive management to establish a partnership and allowing time to build trust; (ii) using scientifically robust study designs with appropriate sample size calculations; (iii) formalising data exchange arrangements and allocating adequate resource for data extraction and re-categorisation; (iv) assessing effects at individual/households level where possible; (v) designing a mixed-methods process evaluation to measure intervention fidelity, dose and unintended consequences; and (vi) ensuring scientific independence through formal contract agreements.

**Conclusions:**

Our collective experiences of working in non-financial partnerships with national supermarket chains could be useful for other research groups looking to develop and implement supermarket studies in an efficient manner. Further evidence from real-life supermarket interventions is necessary to identify sustainable strategies that can improve population diet and maintain necessary commercial outcomes.

## Background

Poor diet constitutes one of the greatest threats to population health, being a significant determinant of overweight, obesity and many noncommunicable diseases [1, 2]. Dietary behaviours are multifaceted and difficult to change, with interventions targeting individually driven behaviour change alone showing limited effectiveness, particularly among populations experiencing disadvantage [3]. Interventions incorporating changes to food environments have great potential to be more effective, equitable and reach many people simultaneously, but are ultimately more challenging to implement, largely due to misalignment of public health and commercial priorities [4].

Supermarkets play a significant role in peoples’ food environments yet their full potential as a setting to improve dietary behaviours has been underutilised. Commercial supermarket chains which are typically a nationally recognised brand, with hundreds of stores sized > 2,000 square feet, each selling tens of thousands of products, hold particular promise for dietary intervention. Such supermarket chains offer a suitable intervention setting for a number of reasons. Firstly, they dominate grocery sales across high income countries, accounting for approximately 83% of all retail food purchases in the United Kingdom (UK), 75% in the Netherlands and 68% in Australia [5–7]. In low and middle income countries supermarkets are also becoming increasingly prevalent [8]. With high numbers of households relying heavily on supermarkets for their food choices and a few companies often owning a large share of total grocery sales, small improvements to the environments of supermarket chain stores could potentially lead to substantial improvements in population diet.

Secondly, supermarkets routinely collect sales data at the store level, and often purchasing data at the household level through loyalty cards, providing objective measures of dietary-related behaviour [9]. Effects of interventions to improve diet have typically been measured with self-reported dietary assessments which are subject to bias, particularly recall and social desirability bias [10]. Purchasing data, however, provide an objective measure of household level dietary-related behaviours and have been shown to provide a reasonably accurate measure of overall dietary quality [11]. Supermarkets, therefore, provide an ideal setting for monitoring detailed, timely, and often inexpensive data on the effects of interventions aiming to improve the healthfulness of food purchases [12]. These large, routinely collected datasets also provide a unique opportunity to apply alternative study design methodologies [13].

Thirdly, customers’ food purchasing behaviours can be examined in a real-life setting. Experimental interventions designed to test a hypothesis under optimal scientific conditions are important to assess proof-of-concept and the efficacy of isolated intervention components [14]. Virtual supermarkets and laboratory settings provide strict and controlled environments that produce high internal validity, but have lower generalizability because they are performed in conditions different from real life (e.g. without children, aromas etc.) [15, 16]. Studies conducted in field settings are needed to understand the extent to which an intervention is effective at changing food purchasing behaviours in real-life circumstances [17]. Field setting studies have high generalizability, but lower internal validity due to the challenges of implementing strong study designs and interventions with high fidelity. A larger number of studies are therefore required to accurately determine intervention effects.

The aim of this paper is to outline the opportunities, challenges and lessons learned from conducting independent evaluations of interventions with national supermarket chains that test the effectiveness of creating healthier in-store environments. These experiences have been distilled into specific recommendations for conducting high quality research with commercial retail chains. The findings aim to provide novel insights for researchers and policy makers hoping to broker similar relationships and contribute to the evidence base in this field.

## Methods

This study adopted a case study approach to generate an in-depth and multi-faceted understanding of working with national, commercial supermarket chains in high income countries in real-life contexts [18]. Collective case study methodology [19] was applied whereby experiences from multiple cases across three high income countries were reviewed simultaneously to generate broader appreciation of the factors to be considered when conducting high quality research with large commercial supermarket chains.

**Fig. 1:**
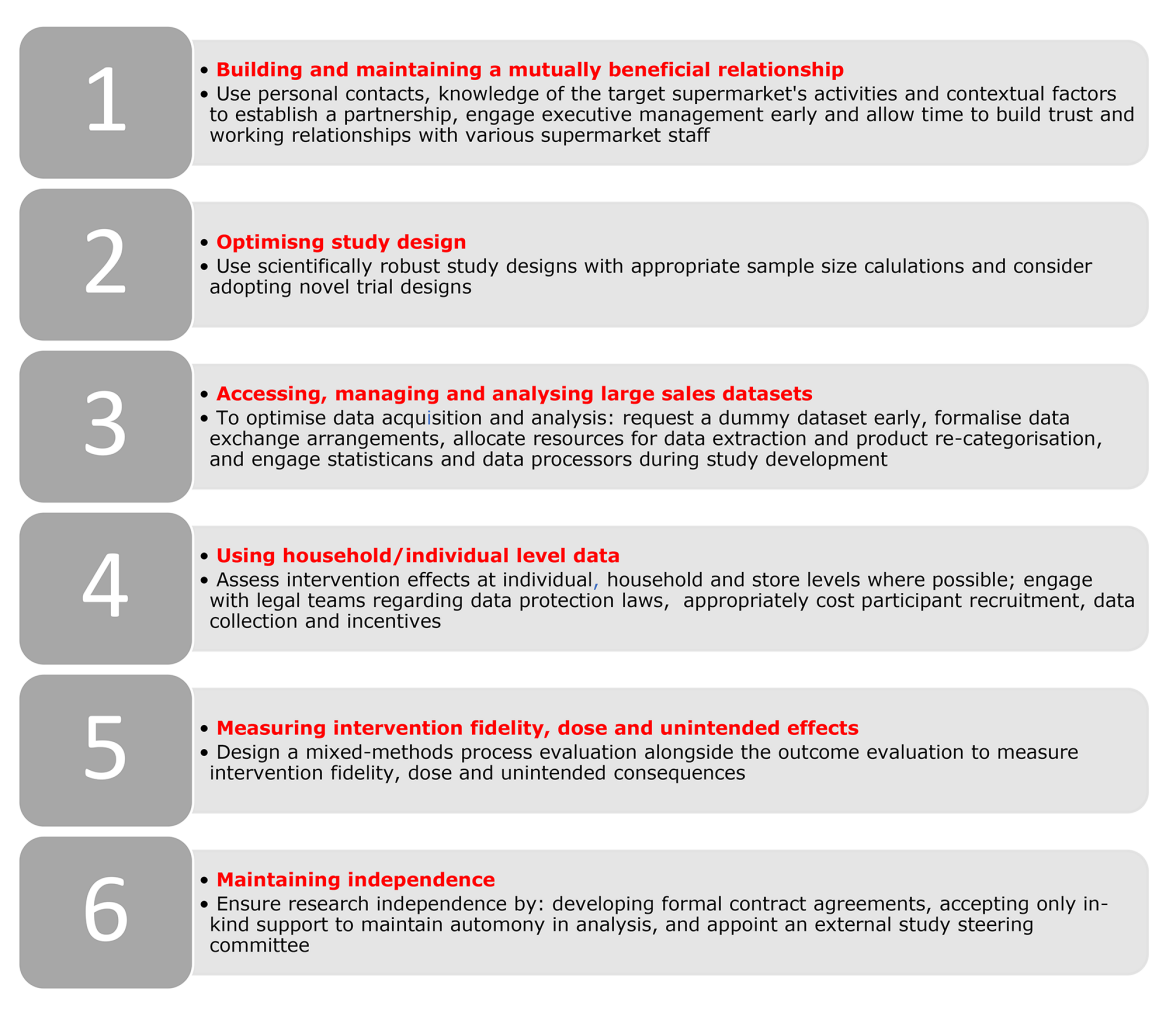
Six recommendations for conducting high quality public health research with national supermarket chains

The collective case study approach applied in this study followed the fours key phases of research activity recommended by Crowe et al. [18]:

1. *Defining the aim of the study* – the specific aim of this study was carefully formulated based on reviews of existing literature (including a number of reviews conducted by the authors of this paper [4, 20, 21]) alongside meetings and symposia with experts in the field acknowledging a knowledge gap in guidance for working with supermarket chains in high income countries.
2. *Selecting the cases* – the selection of research activities included in this collective case study draws from the authors own work which has involved collaborating with major national supermarket chains from market leaders through to smaller nationwide players in three high income countries, namely Australia, the Netherlands and UK. Table 1 provides detailed summaries of each project included in this study. Each case was carefully selected to enable comparisons across different types of: in-store interventions, outcome measures, supermarket chains and target populations. As is recommended when using case study design [18], the researchers knew each selected case well and had peer-reviewed publications related to their cases which provided detailed and mixed-method data to collectively draw upon to answer the aim of this case study design.
3. *Collecting and synthesising the data* – the lead authors (CV, CD) initially complied an extensive list of lessons learned across all cases through face-to-face and video-call discussions. All authors contributed to a process of prioritisation and refinement of the extensive list through video-call and email communications. Each author subsequently detailed their research team’s experiences in relation to the refined list, resulting in a cross-case comparison and analysis for each lesson. This synthesis process identified shared experiences as well as unique challenges and opportunities and enabled all experiences to be distilled into six recommendations for how to conduct high quality research with commercial supermarket chains (Fig. 1).
4. *Reporting and refining the written summaries* – the lead authors (CV, CD) drafted the initial manuscript to characterise our collective experiences and related them to existing literature. The manuscript was refined with input from all authors.

**Table 1:** Summary of studies included in this narrative synthesis

Study name, Country	Study design	Setting	Sample (store & participants)	Intervention description	References
SHELf Australia	RCT	Second largest national supermarket chain	2 stores 574 women	Four study conditions: 1) 20% price-reduction on fruit, vegetables, water and low-calorie beverages 2) Tailored skills-based behaviour-change intervention 3) Combined skill-building and price-reduction 4) Control group Intervention duration: 3 months	Ball et al., 2011, BMC PH Ball et al., 2015, AJCN Olstad et al., 2016, IJBNPA Le et al., 2016, SSM
Healthy Checkout Counter studies The Netherlands	Quasi experimental design (3 experiments)	Largest national supermarket chain	Three study components: 1) 24 stores (15 intervention, 9 control) 2) 2 stores 3) 1 store	Three study components: 1) Increased availability and prominent positioning (check-out counter) of healthier snacks 2) Increased availability and prominent positioning (check-out counter) of healthier snacks in combination with pricing strategies 3) Removal of unhealthy products at check-out counter and replaced with healthier products Intervention duration: 2 months	Huitink et al., 2020, BMC Public Health Huitink et al., 2020, Int. J. Environ. Res. Public Health
Healthy Supermarket Coach studies The Netherlands	Quasi experimental design (2 experiments)	Largest national supermarket chain	4 supermarkets (3 intervention, 1 control) and 4 schools (3 intervention, 1 control)	Study conditions: 1) Peer education workshop for adolescents by supermarket staff 2) Control group Intervention duration: 1 week	Huitink et al., 2021, Health Educ Behav
Social norm nudges in supermarket trolleys The Netherlands	Quasi experimental design	Largest national supermarket chain	1 store	Intervention component: 1) Shopping trolley placemat with a social norm message about vegetable purchases and a designated place to put vegetables Intervention duration: 1 weekend day	Huitink et al., 2020, Appetite
Supreme Nudge The Netherlands	Parallel cluster-RCT	National (cooperative) supermarket chain	12 stores (6 intervention, 6 control) ~ 400 adults aged 30–80 years	Study conditions: 1) Nudging (prominent positioning, labelling, healthy check-outs, healthy baskets, healthy end-of-isles 2) Pricing (decreased prices of healthy foods and increased prices of unhealthy foods) strategies 3) Control group Intervention duration: 12 months	Lakerveld et al., 2018, BMC Public Health Stuber et al., 2020 Nutrition Journal Middel et al., 2021 IJHPM Stuber et al., 2022 Nutrition Journal
WRAPPED (pilot) UK	Prospective matched controlled cluster design	National discount supermarket chain	6 stores (3 intervention, 3 control) 150 women aged 18–45 years	Study conditions: 1) Removal of unhealthy products from checkouts and aisle-ends opposite and replaced with non-food items and water 2) Increased availability and prominent positioning of fruit and vegetables 3) Control group Intervention duration: 6 months	Vogel et al., 2021, Plos Med Shand et al., 2021, SSM Dhuria et al., 2021, BMC PH
WRAPPED (full scale) UK	Prospective matched controlled cluster design	National discount supermarket chain	36 stores (18 intervention, 18 control) 1620 women aged 18–60 years	Study conditions: 1) Increased availability and prominent positioning (front of store) of fresh fruit and vegetables section 2) Control group Intervention duration: 6 months	Vogel et al., 2020, BMJ Open Muir et al., 2023, BMC Medicine

## Results

### Building and maintaining a mutually beneficial relationship

The first challenge to conducting public health research in supermarkets, and a question we are frequently asked, is how did you establish the relationship? From our experiences there is no single or ‘best’ method of developing a connection with a supermarket chain. The time period for engagement and relationship development varied significantly in our studies from several months to many years. Engagement was triggered via (i) a personal connection, where a previous researcher took up a position within the supermarket chain, (ii) the research group leader being approached by the supermarket chain’s health manager and building the relationship over many years, even decades, and (iii) sending a cold-call letter to the Chief Executive Officer of the target supermarket chain. In the case of the personal contact, a route into the company was facilitated by the established trust this individual had with both the research group and the supermarket head-office staff. Having personal connections within the target group or a stakeholder who holds joint academic and health/community service positions is recognised as a key element to success in community-based and translation research because it offers an important conduit for partnership negotiation [22, 23]. The personal contact involved in our study understood the public health and commercial agenda, and facilitated effective communication and mutual agenda setting.

For another of our research groups, the approach from the supermarket’s executive was driven by two key factors. Firstly, the reputations of the research team leader and university were viewed as offering independent, expert scientific knowledge, and status in the area of nutrition. A supermarket’s reputation may benefit if they are seen to be partnering with respected scientists whose skills in quantitative and qualitative evaluation can offer new insights for the company, and evaluations performed by academic researchers are generally trusted more by the public and policy makers than those completed by supermarkets themselves. Findings from community-based research demonstrate how historical reputation can help or hinder relationship development [24]. Having awareness of how research groups and institutions are currently and historically perceived is important when brokering relationships. The second factor driving the approach from the supermarket was the external environment. Increasingly, retailers are being pressured by governments, health and consumer organisations to do more to promote healthy eating [25, 26]. These external political and societal pressures can facilitate a supermarket chain’s willingness to engage with health/nutrition scientists and improve their social corporate responsibility profile [27]. It is probable that these contextual factors prompted a favourable response to our cold-call letters in combination with the approach taken by the research team, which involved using our previous research findings [28–31] and investigation of the supermarket chain’s health-related activities to make a pitch to company executives. Success was facilitated by the executive’s knowledge of other chains testing similar initiatives and willingness for innovation [32]. A review of studies translating health prevention initiatives into novel settings also highlighted that external drivers, alignment with current practice and openness to risk are key components to success [22].

While the process of engagement adopted for each study was unique, similar approaches were used to sustain the relationships and followed the core practices of good partnership [24, 33]. These include: (i) identifying mutually beneficial objectives and co-creating interventions/evaluations, (ii) maintaining open communication to develop trust between partners, and (iii) managing challenges as they emerged. One challenge, experienced in all three settings, was the need to continually realign the relationship as a result of changes in the commercial context, particularly staff turnover. It is well recognised in the field of translational research that the individuals involved play an important role in level of success [34, 35]. We found that certain supermarket staff were particularly motivated to be involved in our research and if they left the company or changed roles it made continuity difficult because they had not been part of the co-design process [36]. Our studies have been achieved by working with staff from multiple departments, including marketing, merchandising, customer relations, analytics, legal, as well as store managers and workers, and executive management. Receiving buy-in from executive management was of utmost importance to each of our studies. This authority was required for all levels of activity, largely because the time commitment involved can draw supermarket staff away from their core responsibilities. Sustained leadership approval is also recognised as key to success in community-based research [24].

Synthesising our experiences and evidence from the field of community-based and translation research, to engage and sustain a successful relationship with a commercial supermarket chain we recommend: (i) using personal connections, knowledge of the supermarket chain’s activities and contextual factors to build partnerships and identify objectives that meet the needs of both parties, (ii) engaging the supermarket’s executive management early and (iii) allowing time to build trust and working relationships with various supermarket staff.

### Optimising study design

Systematic reviews of health-focused supermarket interventions demonstrate that most evidence to date come from studies of poor quality design [14, 37–42]. There is good quality evidence of effectiveness only for price reductions on healthy foods because these studies have low risk of bias due to their randomised control trial (RCT) designs [14, 21, 37, 42]. The randomised or factorial design of price studies is possible because intervention implementation and randomisation can occur at the individual level. Our Supermarket Healthy Eating for Life (SHELf) study in Australia provides one example [43, 44]. In this trial, the independent effects of a 20% price reduction on fruit and vegetables, and healthy eating behaviour change materials, as well as their combined effects, in comparison to a no intervention control were assessed. The intervention was individually targeted because subsidies were applied through participants loyalty cards and behaviour change materials delivered to personal addresses.

Optimal RCT designs, particularly those with appropriate statistical power, are however, considerably more challenging to implement for interventions at the store level. These types of interventions can involve altering the availability or positioning of products, or using shelf or trolley prompts to inform customers of healthier options and require cluster trial designs. The need for power calculations that take account of clustering at the store level has been mostly overlooked in this field of research [20]. While pilot studies are important for building trust between partners and testing the feasibility of intervention components, they do not provide robust scientific evidence. In cluster designed studies, it is the number of clusters, rather than the number of individuals, that are most potent in determining a study’s statistical power [45]. With funding boards often requesting studies have up to 90% power to detect outcome effects, it is important for research teams to engage with statisticians and liaise closely with supermarket staff to obtain support for adequate store numbers in future supermarket intervention studies. The Women’s Responses to Adjusted Product Placement and its Effects on Diet (WRAPPED) study is a cluster design product placement intervention study in the UK with sample size calculations that indicate 16 stores in each arm (32 in total) and 30 participants per store will provide 90% power to detect a conservative change in weekly fruit and vegetables purchased of 1.5 portions [46]. The strength of our study’s design is weakened, however, by the inability to randomise clusters within the company’s business model. Evaluating interventions in commercially competitive, real-world settings frequently necessitates the use of alternative study designs. Similar to other supermarket studies [47–49], the WRAPPED study opted for a quasi-experimental prospective matched-controlled cluster design. Parallel designs with control groups matched on key neighbourhood and store characteristics, and data collected at the same time for each pair of stores, offers a robust study design that increases the similarity of intervention and control stores, and reduces concerns about confounding, such as seasonal shopping trends. While evaluating store-based interventions in real supermarkets can create certain scientific constraints, such as an inability to randomise, these are balanced by the value that such a setting provides an understanding of intervention effectiveness in complex social contexts [50]. Randomisation for store level interventions is possible with strong partnership and close co-creation with the collaborating supermarket as illustrated in our Supreme Nudge cluster-RCT [51].

We recommend that future studies use scientifically robust study designs with appropriate sample size calculations wherever possible. We recognise, however, the challenges researchers can face attempting to realise this goal when working with commercial partners and so recommend consideration of more novel trial methods. Stepped-wedge approaches, for example, lend themselves nicely to store-level evaluations because the intervention can be rolled-out sequentially and rely on regular follow-up assessments (i.e. weekly sales data) [13]. Another advantage is that all stores receive the intervention at some point which could help to motivate store managers to participate. Synthetic controls could be used to evaluate outcomes when there is only one, or a small number of control stores [52], while propensity scores offer an approach to reduce the effect of confounding in real-world supermarket intervention research when randomisation or matching is not possible [53].

### Accessing, managing and analysing large sales datasets

Accessing big data, such as supermarket sales data, for research can be a challenge due to large time and/or cost requirements [54]. Unlike research using costly third-party commercial food datasets [55], our relationships with supermarkets have been non-financial. All of our research groups, however, experienced delays accessing sales data because of drawn-out negotiations or considerable lags to data transfer. In one setting, the supermarket willingly agreed to provide all necessary product-level data, however, once the intervention was completed there were lengthy delays to data acquisition and multiple in-person discussions with the supermarket analytics team were required. In other settings, a dummy sales dataset was provided early during study development which facilitated swift agreement and efficient transfer of the data required for the first phase of the study. Following changes to the supermarket’s executive management, however, data sharing arrangements had to be reviewed and became subject to lengthy contractual negotiations which required clear data visualization, transfer and storage plans to be formally agreed.

Managing the complexity of supermarket sales data has been noted previously by health researchers [9, 56] but few specific examples of issues or tips to ease working with these datasets have been described. From our experiences, data received directly from the supermarkets require a substantial investment of time for reformatting and re-categorisation before statistical analysis can be performed. Food retail datasets are created for commercial purposes and the categories are often not appropriate for health-focused intervention evaluations. For example, retailers may include fresh potatoes in their ambient vegetable category and pre-packed creamy salads in their chilled vegetable category but these products are not included in many national ‘5-a-day’ recommendations and therefore require re-categorisation [49]. Similar efforts were required for our Dutch checkout counter studies to ensure that only products placed at checkouts, not elsewhere in the store, were used to assess intervention effectiveness [48]. In the Supreme Nudge trial we have developed an application programme interface which automatically labelled products as compliant with dietary guidelines or not. Furthermore, the dynamic nature of the food retail sector with its continual product development, requires data cross-checking for the entire study period to identify new items or those no-longer available. Merchandising differences across supermarkets means that efforts to re-categorise products may not be transferrable across studies and differences in the unit of analysis can occur. For example, our Australian studies used grams of fruit and vegetables purchased as the outcome; however, this unit of analysis could not be applied in our UK research because only packaged produce was sold and weights are not specified because suppliers’ quantities vary throughout the year.

Analysing supermarket sales data can also present challenges for which our experiences may help other researchers prepare. The nature of supermarket collaborations often necessitates the protection of commercial sensitivities such as raw sales figures. Using percentage sales figures is one approach (target product sales/total sales) [57], or applying Fisher-Yates transformations [58] of store sales and interpreting the results on the original scale enables change in food portions to be described to demonstrate public health relevance [49]. This approach maintained commercial confidentialities and provided helpful transformations to normality for statistical analysis because stores sales and household loyalty card datasets are often not normally distributed. Datasets can contain many zeros because customers may not shop or buy the same products each week or products may not be available to buy. These skewed data necessitate statistical transformations. Our Australian price study used bootstrapping on loyalty card data to produce robust standard errors and enable linear regression models [44]. In our other studies transformations did not sufficiently improve customer data distributions therefore variables were grouped into tertiles or dichotomised and logistic models performed [59, 60].

Many supermarket intervention studies have applied difference-in-difference analysis methods [14, 41], which collapses data points into a single value for each study period to assess intervention effects. A more sophisticated method, which utilises multiple weekly sales data points from intervention and control groups is controlled interrupted time series (CITS) [61]. We have used CITS to evaluate store sales data in a similar manner to Ejlerskov et al. [62]; models were fitted separately for each pair of stores to account for store matching, followed by random effects meta-analysis [63] to synthesize differences between store pairs at the time of intervention, and 3- and 6-months post-intervention [49]. For some studies data covering particular seasonal periods (i.e. Christmas) may need to be removed or seasonal terms added to the analyses. Engaging a statistician and skilled data processor during study development will ensure data management and analysis issues are dealt with efficiently.

Learning collectively from our experiences, we advocate following five steps for efficient acquisition, management and analysis of supermarket sales data: (i) request a dummy sales dataset early to identify products/categories and all data required for outcome and process evaluations, keeping in mind that product-level details can change over short time frames; (ii) outline up-front and in a formal agreement the data exchange arrangements; (iii) discuss with supermarket staff the time needed to extract, prepare and transfer data to the research team, acknowledging that research projects are not core supermarket business; (iv) allow researcher time and resource for re-categorisation of products in vast datasets; and (v) involve a statistician and skilled data processor to facilitate data transformations and analyses to make best use of the available data.

### Using household/individual level data

As public health researchers we are intrinsically interested in understanding intervention effects at the population level. Store-level sales data enable population evaluation because they are objective and generalizable to all customers of participating stores over the study period [64]. Increasingly these data are being used as the primary outcome in supermarket intervention studies, such as our Dutch healthy checkout counter studies [48, 65] and other research [41, 42]. However, store-level data cannot identify if changes in product sales are attributable to changes in existing customers’ behaviours or to new customers coming into the store. Assessment of outcomes at the household or individual level are required to understand who responds to supermarket interventions and whether such interventions address or increase dietary inequalities [21, 66]. Our studies have used loyalty card, receipt, dietary and health data from participants in addition to store sales to assess changes at individual, household and population levels [49, 59, 60, 67].

While the decision to recruit participants to assess individual/household level data is scientifically important, there are resource and time implications, and data protection obstacles to consider. Data protection laws can create an extra layer of complexity to recruit participants and access their loyalty card data because customers own, and supermarkets host, these data. In some of our studies, potential participants had to be identified and invited by the supermarkets in order to comply with data protection laws. Interested participants then contacted the research team directly and this approach had varying levels of success [44, 46]. The SHELf study reached sample size estimates much faster than expected, while the WRAPPED study required in-store recruitment to boost participant numbers. Loyalty card data sharing can also require participants to provide traceable consent to the supermarket which provided an additional hurdle to our research teams accessing these data. Being aware of national/regional data protection regulations and their interpretation by large retailers who risk substantial fines. Reputation damage is important when developing supermarket intervention studies and requires engagement with organisational legal teams. Alternative methods to recruit individuals which overcome data protection agreements include sending invitation letters to all residents located around participating supermarkets or local marketing strategies as applied in our Supreme Nudge study [51].

Additional resource and time costs required for individual/household level data can include the provision of incentives to encourage participant recruitment and retention. Our studies included incentives of up to £30/$AUD60/€40 in vouchers, cash or groceries [43, 46, 51], but higher values may be necessary to compete with market research companies. The time costs for supermarket staff to recruit participants and extract customer data can be a potential barrier. We found, however, that this barrier could be counter-balanced by mutual interests in identifying differential intervention effects by customer characteristics (age, sex, income etc.). Additional researcher resources are necessary to collect purchasing, dietary or health outcome data in some studies. Our Supermarket Coach study, which aimed to improve food choices among adolescents in Dutch supermarkets, collected receipts from participants at three time points. This approach was adopted because few young people hold loyalty cards and it provided more objective data to assess intervention effectiveness [68, 69]. Several of our studies collected individual level data alongside loyalty card or store data [44, 46, 51, 60]. Our Supreme Nudge trial collected information about diet, biomarkers and anthropometrics [51], and the WRAPPED study collected data about diet from one or more household member, food waste and psychosocial factors [46]. Analyses of these variables will offer important insights into health effects, possible mediators, and the validity of loyalty card data as an outcome measure [54].

To build the evidence base in this field, we recommend future research assesses effects at the individual and household level. We recognise data protection laws, time and resource constraints can hinder collection of these data, but our experiences demonstrate that by engaging with organisational legal teams and appropriately costing and resourcing study activities, high quality individual and household level data can be collected to improve understanding of how different customer groups respond to supermarket interventions.

### Measuring intervention fidelity, dose and unintended effects

Given the complex nature of conducting supermarket research in a real life context it is paramount to complete process evaluation activities alongside outcome evaluations; yet such evaluations have rarely been reported [70–72]. There is a number of models and frameworks which can be adopted including the MRC process evaluation guidance, RE-AIM framework, PRISM model and system science approaches [73–76]. For commercial supermarket studies it is important to measure the (i) alignment of intervention implementation with the protocol, (ii) context and dose of the intervention and (iii) individuals’ experiences of the study (supermarket staff and participants/customers). Collecting these data will provide crucial details to understand evaluation outcomes, improve the design and execution of future studies, and help translation into commercial practice across different contexts.

Assessing whether an intervention has been carried out as intended and capturing deviations from the protocol is particularly important for supermarket studies that involve alterations to the in-store environment because they are often implemented by numerous supermarket staff. Similarly to previous US research [71], in-store assessments pre- and post-intervention have been conducted as part of the WRAPPED study process evaluation activities. Assessments include pre-existing in-store assessment tools [28, 77], photographs, plus a bespoke survey completed with store managers four times over the study period [46]. In our Supreme Nudge trial ‘Reflexive Monitoring in Action’ approach was applied allowing implementation problems to be identified through biweekly or monthly checks and discussed with involved staff so that implementation strategies could be adapted in real-time [78]. In-store assessments, however, are resource intense [48] and alternative approaches may need to be adopted. For example, our Dutch Healthy Checkout Counter and WRAPPED studies used sales data and store planograms to scrutinise fidelity. These desk-based investigations and discussions with store staff identified instances when products were inappropriately positioned and this was accounted for statistically through sensitivity analyses.

For individually targeted interventions, such as price subsidies or healthier swaps, data about intervention dose can be collected from participants. Our SHELf study used both quantitative and qualitative methods to obtain information that could aid interpretation of the outcome results [70]. For example, many participants felt discounts on water and low-calorie soft drinks were of little use because they never buy these products, while others did not use the fruit and vegetable subsidy because the quality or cost of fruit and vegetables was considered better in competing stores. Assessing the study context can also aid measurement of intervention dose or exposure or mechanism of impact. Our WRAPPED study collected details of all retail food outlets used by participants and approximate spend in each store in an effort to quantify intervention dose [46] and assesses social influences on shopping behaviours [79]. Furthermore, assessment of the broader commercial and policy context was conducted to help inform the translation of findings and potential diffusion of innovation in new practices across the supermarket sector [80].

Process evaluation activities can also provide information on unintended effects. Few studies have used sales or purchasing data to assess substitution effects yet such analyses can indicate intervention impact on overall shopping patterns. Our economic evaluation for SHELf showed small unexpected substitution effects among participants in the price subsidy arms where purchases of bakery and dairy products increased alongside fruit and vegetables [81]. Qualitative research can reveal information about staff and participant experiences. Interviews with supermarket staff in the Dutch Healthy Supermarket Coach study identified an unexpected benefit. Several staff trained to implement the intervention were subsequently promoted because they had demonstrated competence in a more responsible role. Interviews with participants of SHELf and WRAPPED showed personal benefits from engaging in the studies, in particular receiving financial reward and supporting research that could benefit society [82]. These outcomes were advantageous for the supermarket chain and research team and lead to larger-scale implementation of the interventions and other opportunities [67].

We recommend designing a mixed-method process evaluation alongside the outcome evaluation to measure intervention fidelity, dose and unintended consequences. Contextual factors should also be assessed to ensure understanding of which approaches worked best in certain contexts and what support may be needed to ensure success. The extent of the process and implementation evaluation will depend on available resources, but these activities should be prioritised where possible to provide important information for interpreting outcome findings and translating evidence into policy and practice.

### Maintaining independence

There has been much debate in the field of public health nutrition about the real and perceived conflicts of interest that can result from public sector population health researchers working with food industry [83–85]. In an effort to minimise risk of our research teams’ credibility being undermined and to uphold scientific integrity we adopted strategies in our collaborations with supermarket chains to ensure independence and accuracy of our research results. Key consistencies across our studies included accepting only in-kind support from our partnering supermarket chains, and each university negotiating and signing contractual agreements up-front to govern the collaboration. These contractual discussions can be lengthy and required engagement of legal teams, and compromise from both sides. Agreements were different in each setting, including Memorandums of Understanding, initial non-disclosure agreements, Collaborative Research Agreements and/or data agreements. The contracts covered similar content, namely clear summary of the work (e.g. project aims, study design, anticipated intervention content and implementation, and analysis plans), each partner’s roles and responsibilities, data ownership and sharing arrangements, plus confidentiality and publicity boundaries. Incorporating these specific details in signed agreements provided security and reassurance for each partner. We found it was important to be transparent about schedules, processes and publicity timelines because of the time lag between project design and scientific publication of the results; this timeframe can be at odds with the fast moving pace of activities in the supermarket sector.

Accepting only in-kind support from the collaborating supermarkets afforded a number of benefits for our research teams, particularly independence in analysis and greater external trust in our studies’ findings. The in-kind support from supermarkets largely took the form of implementing the interventions (including staffing, products and equipment), plus the collection, extraction and sharing of store sales and loyalty card data. Ensuring independent analysis and reporting of study data was paramount for each research team. Liaison with supermarket staff was required in some instances to ensure accuracy in understanding and re-categorising sales data, and to achieve joint publicity ventures. Independence was further ensured through study steering committee oversight. Committee members were external to the research team and supermarket chain, and included representatives from regional municipalities, health boards, non-government organisations and reputable universities. They regularly reviewed study activities and provided valuable expertise and input regarding balancing private commercial interests against public health benefits.

We recommend research groups maintain independence from the supermarket chain by following three key strategies: (i) develop formal contracts involving organisational legal teams and clear summaries of the work and reporting arrangements, (ii) accept only in-kind support to maintain autonomy in analysis and (iii) appoint an external study steering committee to regularly monitor study activities.

## Discussion

This paper describes the experiences and lessons learned from a number of different trials our research teams have evaluated with national supermarket chains in Australia, the Netherlands and UK. While our analysis is unique in its application to national, commercial supermarket chains, research teams from the US and UK have previously synthesised their experiences of working with small independent grocery and convenience stores to test healthy eating interventions [86, 87]. There are some key similarities to note when working with the food retail sector spanning both supermarkets and small stores. In particular, the need to: (i) allocate adequate time to build trusting and mutually beneficial relationships, (ii) minimise the work and effort required from retailers to implement interventions and/or collect/extract evaluation data, and (iii) recognise the challenges accessing and manipulating sales data. Additionally, supermarkets and convenience stores’ practices are driven by commercial outcomes which need to be negotiated and respected when designing interventions, evaluations and dissemination activities.

A key difference between our case study synthesis and previous examples from the convenience store sector relates primarily to our emphasis on robust scientific study designs, alongside comprehensive mixed-methods evaluations. This emphasis in our recommendation is appropriate for partnerships with commercial supermarket chains because of the routine data that are collected at store and households levels, and because of the large number of stores available to achieve adequately powered trials. Working with independent grocery and convenience stores requires greater negotiation and relationship building at the store level which increases the time and challenges of achieving optimal sample size calculations, particularly when cultural and linguistic diversity is considered [86]. Another key difference to be considered when working with large supermarket chains is the need for independence and contractual negotiations which are of great importance to both commercial and academic institutions as outlined above under recommendation six.

This study provides unique insight into the challenges and opportunities of conducting research partnerships with commercial supermarket chains who have nationwide reach into the shopping trolleys of millions of families. We adopted a case study approach which covered studies in three high income countries. This approach may reflect experiences unique to these particular research studies and contexts which could be considered to limit generalisation of our findings. The four-stage process for data synthesis used in this study, however, identified six cross-cutting themes that were applicable to each of the real-world supermarket trials, contexts and countries examined. It is possible that alternative challenges and opportunities working with supermarkets to conduct public health nutrition trials could arise in different settings.

## Conclusion

Supermarkets are an important setting to influence dietary behaviour. Even small changes in food purchasing can shift population level dietary intake in a healthier direction. We believe that the six recommendation and synthesis of lessons we have learned from working with national supermarket chains can help guide the development and implementation of more and scientifically robust supermarket intervention studies. Further evidence from real-life supermarket interventions will help to identify sustainable strategies that can improve population diet and maintain necessary commercial outcomes.

## Data Availability

Not applicable as no new empirical data were collected.

## Abbreviations

CITS: Controlled Interrupted Time Series
SHELf: Supermarket Healthy Eating for Life
UK: United Kingdom
WRAPPED: Women’s Responses to Adjusted Product Placement and its Effects on Diet
